# A Multidisciplinary Review of the Roles of Cripto in the Scientific Literature Through a Bibliometric Analysis of its Biological Roles

**DOI:** 10.3390/cancers12061480

**Published:** 2020-06-05

**Authors:** Elisa Rodrigues Sousa, Eugenio Zoni, Sofia Karkampouna, Federico La Manna, Peter C. Gray, Marta De Menna, Marianna Kruithof-de Julio

**Affiliations:** 1 Department for Biomedical Research, Urology Research Laboratory, University of Bern, 3008 Bern, Switzerland; elisa.rodriguessousa@dbmr.unibe.ch (E.R.S.); eugenio.zoni@dbmr.unibe.ch (E.Z.); sofia.karkampouna@dbmr.unibe.ch (S.K.); federico.lamanna@dbmr.unibe.ch (F.L.M.); marta.demenna@dbmr.unibe.ch (M.D.M.); 2 Department of Urology, Inselspital, Bern University Hospital, 3010 Bern, Switzerland; 3 Department of Urology, Leiden University Medical Center, 2333 ZA Leiden, The Netherlands; 4 Biotheranostics, Inc., San Diego, CA 92121, USA; pgray816@gmail.com

**Keywords:** CRIPTO, TDGF-1, bibliometric analysis, cancer, development, biochemistry and molecular biology, experimental medical research

## Abstract

Cripto is a small glycosylphosphatidylinisitol (GPI)-anchored and secreted oncofetal protein that plays important roles in regulating normal physiological processes, including stem cell differentiation, embryonal development, and tissue growth and remodeling, as well as pathological processes such as tumor initiation and progression. Cripto functions as a co-receptor for TGF-β ligands such as Nodal, GDF1, and GDF3. Soluble and secreted forms of Cripto also exhibit growth factor-like activity and activate SRC/MAPK/PI3K/AKT pathways. Glucose-Regulated Protein 78 kDa (GRP78) binds Cripto at the cell surface and has been shown to be required for Cripto signaling via both TGF-β and SRC/MAPK/PI3K/AKT pathways. To provide a comprehensive overview of the scientific literature related to Cripto, we performed, for the first time, a bibliometric analysis of the biological roles of Cripto as reported in the scientific literature covering the last 10 years. We present different fields of knowledge in comprehensive areas of research on Cripto, ranging from basic to translational research, using a keyword-driven approach. Our ultimate aim is to aid the scientific community in conducting targeted research by identifying areas where research has been conducted so far and, perhaps more importantly, where critical knowledge is still missing.

## 1. Introduction

Cripto (TDGF1, CRIPTO-1) is a small glycosylphosphatidylinisitol (GPI)-anchored and secreted oncofetal protein that plays important roles in regulating stem cell differentiation, embryogenesis, and tissue growth and remodeling [1]. When aberrantly expressed, Cripto can drive tumor initiation and progression in multiple tumor types, including prostate cancer [2], breast cancer [3], colon cancer [4], hepatocellular carcinoma [5], glioblastoma [6], and lung cancer [7]. Cripto modulates the signaling activity of multiple TGF-β superfamily ligands, including activins and TGF-β1 [8,9,10,11,12], and is an essential coreceptor for others, including Nodal, GDF1, and GDF3 [13,14,15]. When released from cells in a soluble form, Cripto can act in a growth factor-like manner to activate SRC/MAPK/PI3K/AKT pathways. Although the receptor mechanism involved in this growth factor-like signaling has not been clearly established, it has been shown to occur in a TGF-β-independent manner [12]. Notably, Cripto binds to cell surface GRP78 and this interaction is critical for Cripto signaling via both TGF-β and SRC/MAPK/PI3K/AKT pathways [16]. Cripto has also been shown to regulate additional signaling pathways involved in cell differentiation and development, such as Wnt and Notch [17]. Therefore, Cripto interacts with multiple signaling pathways that play key roles in regulating normal tissue homeostasis and tumorigenesis. However, its molecular mechanisms and signaling partners remain only partially elucidated.

The use of bibliometric methods is a novel approach to identifying and summarizing literature on a selected topic in a systematic manner and to identifying patterns in a research area. Bibliometrics have been applied to several different research fields [18,19,20]. In the last ten years, multiple bibliometric software tools have been developed, including CitNetExplorer [21], CiteSpace [22], HistCite [18], VOSviewer [23], and Bibliometrix [24]. 

## 2. Results

### 2.1. Bibliometric Analysis

**Figure 1:**
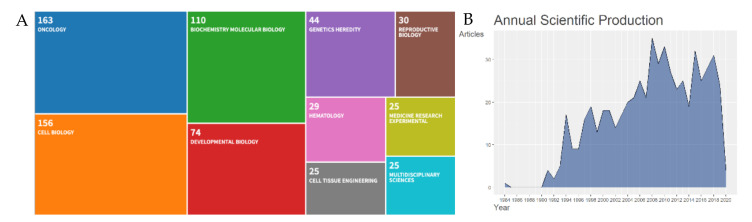
(**A**) Web of Science (WoS) categories. Visual representation of classification methods for scientific literature on Cripto research. Ten fields of knowledge were retrieved from the WoS repository: Oncology (*n* = 163), biochemistry and molecular biology (*n* = 110), genetics heredity (*n* = 44), reproductive biology (*n* = 30), cell biology (*n* = 156), developmental biology (*n* = 74), hematology (*n* = 29), medicine research experimental (*n* = 25), cell tissue engineering (*n* = 25), and multidisciplinary sciences (*n* = 25); (**B**) annual scientific production on Cripto research. Growth of the annual number of documents in the Cripto research field from 1900 to 9th April 2020.

The systematic approach we undertook helped to curate the Cripto research fields into logical categories. We performed a temporal screening and showed that Cripto has received increased attention and interest by researchers over the last two decades. For the last 10 years, a total of 271 documents fit the search criteria across eight document types (DT). The eight document types were articles (*n* = 212), editorial material (*n* = 1), letters (*n* = 3), meeting abstracts (*n* = 40), reviews (*n* = 14), and a review-book chapter (*n* = 1), and the most three relevant sources were Cancer Research (*n* = 13), PLoS ONE (*n* = 9), and Development (*n* = 8). A further breakdown of the research was based on the following ten WoS categories: oncology (*n* = 163), cell biology (*n* = 156), biochemistry molecular biology (*n* = 110), developmental biology (*n* = 74), genetics heredity (*n* = 44), reproductive biology (*n* = 30), hematology (*n* = 29), cell tissue engineering (*n* = 25), medicine research experimental (*n* = 25), and multidisciplinary sciences (*n* = 25) (Figure 1A). The research content of each category was further organized into more comprehensive branches of disciplines, in order to combine the research work and form more structured categories.

Cripto was described for the first time in the scientific literature in 1989, as a gene of the ’EGF family’ expressed in undifferentiated human NTERA2 teratocarcinoma cells [25], and then further described in 1991 for its role in transforming a normal mouse mammary epithelial-cell line in vitro [26]. After this first characterization, the annual volume of literature increased with a percentage growth rate of 3.92 until 2020 (Figure 1B). 

**Figure 2:**
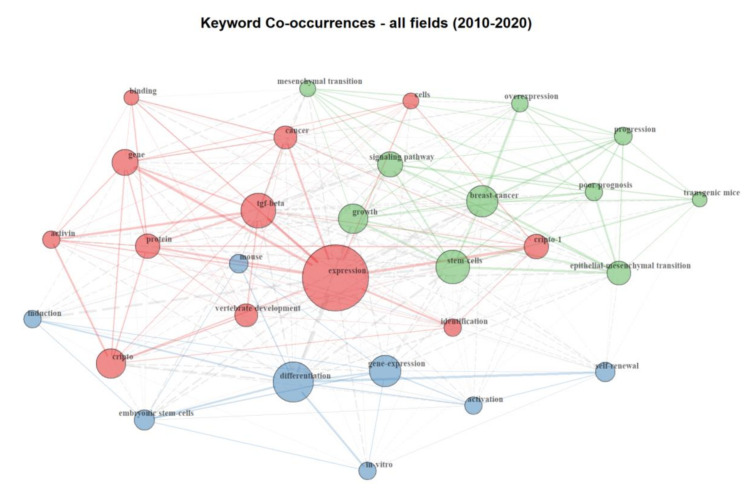
Keyword co-occurrence network. Graphic visualization of co-occurrence interactions between keywords present within documents published between 2010 and 2020 in the Cripto research field. Each keyword is represented as a node and the co-occurring frequency of the keywords appeared determines the circle size. Each co-occurrence of a pair of words is represented as a link, which has a different thickness, depending on the number of times that a pair of words co-occurs in multiple articles. Three main clusters are identified: an embryogenesis cluster (blue), functional biology and development cluster (red), and cancer progression cluster (green).

The bibliometric analysis on Cripto literature, from 2010 and 2020, highlighted 271 documents. Our results show that the keyword co-occurrence network includes three major clusters (represented by different colors) interconnected to each other. The keywords “cripto”, “cripto-1”, and “cancer” are in the same cluster, meaning they are more likely to reflect identical topics (Figure 2). We were able to identify an “embryogenesis” cluster (shown in blue), where keywords such as “embryonic stem cells”, “differentiation”, and “mouse” are highly represented, highlighting the presence of a research branch mainly focused on embryogenesis and animal models. The red cluster, dominated by keywords such as “expression”, “Tgf-β”, and “Cripto”, identifies a branch mainly focused on functional biology and development. Finally, the green cluster, which includes keywords such as “epithelial to mesenchymal transition”, “progression”, “overexpression”, “stem cells”, and “breast cancer”, indicates a branch of the field focused on the involvement of Cripto in cancer progression. Importantly, the localization of the nodes of each cluster and the proximity of the nodes of one cluster with nodes of an adjacent one reflect the connection between areas of research. For example, despite belonging to different clusters, the keywords “cancer” and “mesenchymal transition” are close to each other and highlight the well-known relevance of Cripto in epithelial to mesenchymal transition (EMT) in cancer and cancer progression. Similarly, the keywords “Tgf-β” and “growth” are also close to each other, indicating that research conducted on Cripto and Tgf-β in this field is probably linked to a positive role of these two pathways in development and oncology. Furthermore, the dimension and localization of the keyword “expression” as a central node draws attention to the importance of Cripto expression in all clusters. This highlights the key features of this protein, which is mainly expressed during development and then mostly absent in adult, well-differentiated tissues, but re-expressed during carcinogenesis and inflammation [1].

Interestingly, its overexpression in a number of different types of human carcinomas makes it an attractive therapeutic target. As depicted in the keyword co-occurrence network, words such as “overexpression”, “breast cancer” (both top right), and “binding” (top left) are on opposite sites, suggesting that further development of the potential role of Cripto as a target for cancer therapy will probably occur in the future, as the fields are not yet merged together. In contrast to the localization of the keyword “expression”, the keyword “progression” is located on the top right, without an evident connection to the embryogenesis and development clusters, reinforcing the notion that an overexpression or a re-expression of Cripto has been associated with cancer progression. In conclusion, although Cripto has been defined as a crucial protein in embryogenesis and oncogenesis, further studies regarding its low-level expression in adult tissues, as well as its involvement in stem cell niches and stem cell maintenance, are needed. The keyword frequency has also been schematically represented by keyword wordcloud analysis, and “expression”, “stem cells”, and “differentiation have been shown to be the most frequent keywords used in Cripto research publications (Figure S1).

### 2.2. Cripto in Developmental Biology

Multiple cell lineages (the epiblast, the primitive endoderm, and the trophectoderm) and future body axes (e.g., anterior-posterior (AP)) are formed during mouse embryogenesis [27,28,29,30]. Nodal, a ligand of the transforming growth factor-beta (TGF-β) family, requires the activity of epidermal growth factor-Cripto-FRL1-Cryptic (EGF-CFC) co-receptors; Cripto is required for early Nodal functions, prior to and including gastrulation, whereas Cryptic is required for later Nodal requirements in left–right specification [13,31,32,33]. Cripto characterization as an early epiblast marker and a key extracellular determinant of the naïve and primed pluripotent states was also confirmed in a study focused on the extrinsic regulation of developmental plasticity [34]. Similar findings have also shown Cripto to be expressed from the early blastocyst, embryonic day 5/6 (E5/6), until E11/12 in porcine models [35] and cattle embryos [36].

By modulating Nodal and its downstream signaling mediator Smad2, Cripto can maintain mouse epiblast stem cell (EpiSC) and human embryonic stem cell (hESC) pluripotency [37]. However, a Nodal-independent role of Cripto in the extraembryonic endoderm has also been demonstrated [38]. Furthermore, it is likely that primitive endoderm cells can receive Nodal signaling given Cryptic expression in PrE19, and due to the fact that Cripto and Cryptic can act non-cell-autonomously [39]. Cryptic double mutants display severe defects in the epiblast, extraembryonic ectoderm, and anterior visceral endoderm (AVE), resulting in phenotypes that are highly similar to those of Nodal null mutants [40]. The AP axis is established when the AVE is formed from the distal visceral endoderm (DVE) on the future anterior side of the embryo by embryonic day E6.5. Cripto is required in the epiblast for the migration of the AVE [41]. Although initially expressed in the entire epiblast, Nodal and Cripto expression is restricted to the posterior epiblast during AVE formation, together with posterior genes (e.g., Wnt3 and Brachyury), generating definitive mesoendoderm and forming a primitive streak [42,43,44,45,46,47]. The loss of Cripto in mice dramatically reduces, but does not completely abolish, Nodal activity, leading to the absence of a morphological primitive streak [48]. Cripto-null mouse embryos also exhibit striking defects in mesoderm formation and AP patterning, resulting in early embryonic lethality and the concomitant absence of cardiac marker gene expression [7,46]. Interestingly, mutations in Cripto are associated with isolated congenital heart defects, including conotruncal alignment defects and membranous ventricular septal defects [49], suggesting a role for Cripto signaling in cardiac development. This role was also identified by a study in which the ectopic expression of Cripto in transgenic mouse embryos caused hemorrhages, fatal cardiac defects, and embryonic lethality [50]. In line with this, Nodal/Cripto signaling was described to be involved in the cardiac development of chick embryos [51]. The expression of ectopic isthmin1 (ISM1), a fibroblast group factor that inhibits Nodal signaling, has been shown to cause left-right asymmetry and an abnormal heart position [51]. Defects in the formation of the AP axis, which are a common feature of Cripto knockout embryos, were also measured in a homozygous post-glycosylphosphatidylinositol attachment to proteins 6 (PGAP6) knockout mouse model. In fact, it has been shown that Cripto is a highly sensitive substrate of PGAP6, which plays a critical role in modulating Nodal signaling through the regulation of Cripto shedding [52]. A secretome analysis of mouse embryonic stem cells (mESCs) during cardiac and neural differentiation confirmed that Cripto is linked to cardiac differentiation and that its absence promotes neurogenesis [53]. Moreover, Lefty-1 and Cerberus (Cer-1), two Nodal interactors, have been identified as key secreted elements that discriminate between cardiac and neural differentiation. Lefty, an antagonist of Nodal signaling, was detected in both secretomes in undifferentiated cells and downregulated during differentiation. Cer-1, on the other hand, was absent in an undifferentiated state and was only strongly upregulated during cardiac differentiation [53].

Signals that require an EGF-CFC-family co-receptor (Nodal-like signaling) have been found to play an essential role during several stages of vertebrate mesoderm formation and patterning [42,54,55,56,57,58,59]. An intermediate mesoderm (IM) that gives rise to all kidney tissue is regulated by Nodal-like Cripto-dependent signaling [60].

Cripto expression has been linked to chromosomal instability in a study showing that pristine hESC expressing Cripto are more prone to have, in vitro, a supernumerary number of centrosomes and may thus be prone to develop abnormal multipolar mitoses [61]. The inhibition of Cripto signaling by chemical disruption or an anti-Cripto blocking antibody resulted in a decrease in the number of supernumerary centrosomes in hESC. Taken together, Cripto was considered to be a candidate factor affecting the genomic stability of hESC [61].

### 2.3. Cripto in Reproductive Biology

Studies of cattle have indicated that Cripto is a determining factor of reproduction. In particular, Cripto is expressed by bovine oviduct epithelial cells, as well as by the embryo [62]. Different bovine preimplantation stages in vitro (IVF) and in vivo-derived embryos were compared to study the temporal gene expression dynamics as the embryo develops from a spherical blastocyst on E7 to an ovoid conceptus on E13. Cripto was among the top upregulated genes on E13, suggesting its role in the regulation of implantation conceptus elongation and embryo survival [63].

In human endometrial remodeling during the menstrual cycle, Cripto and Nodal are highly expressed in epithelial and stromal endometrial cells during the proliferative phase, whereas Lefty is undetectable [64]. Cripto is not associated with endometrial pathologies such as adenomyosis (endometrioma-like disease) [65] and endometrioma, considering that protein Cripto levels and downstream activation of the SMAD3/4 pathway were indifferent in endometriosis cases compared to control endometrial tissues [66]. Interestingly, no association of Cripto with endometriosis or endometriosis-related endometrial cancer was shown; however, the deregulation of Cripto was found in endometrioid ovarian cancer [67]. Imbalanced levels of Cripto and Nodal in the placenta led to aberrant Activin signaling activation, resulting in an excessive proinflammatory effect associated with pathologic preterm delivery [68]. Cripto expression has also been investigated in an abnormally adherent placenta (creta placenta). A correlation between the expression of Cripto and an immature state of the trophoblast that led to an increased invasion and adherence of the placenta to the uterus was observed [69]. In conclusion, the regulation of Cripto expression levels is essential for normal embryogenesis, while its deregulation may contribute to certain pathological conditions of the reproductive system.

### 2.4. Cripto Signaling in Biochemistry and Molecular Biology 

The multitude of upstream and downstream Cripto interactors creates a complex and finely tuned signaling network. Cripto was initially identified as a modulator of the TGF-β pathway. Interestingly, Cripto can both activate and inhibit the TGF-β pathway, depending on which TGF-β superfamily ligand and signaling receptor are involved [14,31,70]. As a co-receptor for Nodal, GDF1, and GDF3, Cripto forms a complex with activin, activin type II (ActRII/IIb), and activin-like type I (ALK4/7) receptors, leading to phosphorylation of the intracellular signaling mediators Smad2 and Smad3. Once phosphorylated, Smad2 and Smad3 bind to Smad4 and translocate to the nucleus, where they regulate the transcription of specific target genes. In contrast, Cripto also inhibits Activin A, Activin B, and TGF-β1 signaling by forming complexes with these ligands and their respective signaling receptors. Interestingly, given that myostatin uses activin/TGF-β pathway signaling, studies on the Cripto inhibitory potential of myostatin signaling were performed, showing that myostatin’s response was inhibited by Cripto [71]. Moreover, there are reports that suggest that Cripto can also act through Src, MAPK, and PI3K pathways independent from TGF-β pathways and that it may also regulate additional signaling pathways, such as those of Wnt and Notch. 

In 2008, the glucose-regulated protein GRP78 was identified as a new Cripto signaling partner [16]. GRP78 is a master regulator of the endoplasmic reticulum (ER) stress response that can also translocate to the cell surface, where it mediates a variety of signaling responses. A molecular dynamic simulation of the Cripto/GRP78 protein complex showed that Cripto binding locks GRP78 on the membrane, thus possibly enabling the tumorigenic phenotype [72]. Cripto binding to cell surface GRP78 is required for its ability to interfere with TGF-β signaling, but perhaps more interestingly, there is also evidence suggesting that Cripto/GRP78 binding is essential for the Cripto-dependent modulation of Src, MAPK, and PI3K pathways [11].

At a transcriptional level, the Cripto promoter has been shown to contain Tcf/Lef, Smad, Oct4, SNAIL, and hypoxia responsive binding elements [37,73,74,75]. The nuclear receptors liver receptor homolog 1 (LRH-1) and germ cell nuclear receptor (GCNF) have also been identified as transcriptional regulators of Cripto expression. Specifically, LRH-1 positively regulates Cripto expression by binding a DR0 element within the Cripto promoter, while GCNF acts as a negative regulator in both embryonal carcinoma cells and breast cancer cells [76]. In glioblastoma cells, the transcription factor SOX3 induces Cripto expression and promotes proliferation, invasion and migration, possibly via the long non-coding RNA SOX2OT, which targets miR-194-5p and miR-122 and results in Cripto expression [77]. In glioblastoma cells (U87 MG), a positive feedback loop was also reported, in which Cripto regulates its own expression through ALK4/SMAD2/3 signaling [78]. 

Examples of direct and indirect micro-RNA-mediated post-transcriptional regulation of Cripto expression have also been reported. miR-15a-16, miR-15b, and Cripto expression have been shown to be inversely correlated in non-small lung cancer (NSCLC) [79] and glioma cells [80], whereas let-7 microRNA family members, such as LIN28B and TGFBR1, indirectly modulate Cripto expression by targeting known regulators of stem cell pluripotency maintenance [81].

An unbiased screen for novel Cripto binding proteins using a non-transformed human mammary epithelial cell line (MCF10A) expressing a Flag-Cripto protein identified Myosin 9 (MYH9), a member of the Myosin II complex, as a Cripto interactor. The treatment of MCF10A Flag-Cripto cells with Myosin II inhibitor impaired Cripto localization to the cell membrane and its release in the culture media [82]. 

Cripto has been highlighted as a regulator of skeletal muscle regeneration and satellite cell differentiation by antagonizing myostatin signaling [83]. Interestingly, it was also reported that myostatin signaling depends on Cripto and ALK4 in a cell-type-specific manner [84]. 

Recently, a novel role for Cripto that links muscle regeneration and macrophage (MP) plasticity was found [85]. During muscle regeneration, the balance between pro-inflammatory and anti-inflammatory MPs is relevant. Cripto is mostly expressed in the anti-inflammatory MPs. Myeloid-specific Cripto knockout mice (Cripto^My-LOF^) were used to investigate the physiological role of Cripto in the infiltrating MPs, without interfering with its activity in the myogenic compartment. Infiltrating MPs were able to access the muscle, but failed to properly expand as anti-inflammatory CD206+ MPs in both acute injured muscle and Duchenne muscular dystrophy models [85]. It is noteworthy that this reduction in the plasticity of MPs in Cripto^My-LOF^ mice also affects vascular remodeling by modulating endothelial to mesenchymal transition.

### 2.5. Cripto in Oncology 

Adult tissues display negligible Cripto expression levels, while elevated Cripto levels, in situ or in circulation, are found in many human tumors (e.g., breast, colon, prostate, cervix, gastric, and hepatocellular carcinoma) [76,86]. The reactivation of Cripto has been associated with tumorigenesis and although genomic aberrations in the Cripto gene itself have not specifically been reported in human cancers, aberrantly high levels of Cripto expression or experimental overexpression have oncogenic effects, such as the promotion of EMT, angiogenesis, and increased cell proliferation and migration in in vitro and in vivo experimental models [1]. Specifically, in glioblastoma, high Cripto transcript levels, as opposed to low Nodal and Lefty levels, were identified in human patient-derived xenograft models of aggressive glioblastoma, as well as in a human tissue microarray (TMA) of primary cases. A moderate to strong expression of Cripto was found in more than 50% of primary cases and linked to worse overall survival in the younger age groups compared to patients within the same age groups who demonstrated lower Cripto expression [87]. Another study showed that high Cripto expression in glioma coincided with a low expression of miR15b, which was also associated with a shorter survival rate. This negative association was due to the direct inhibition of Cripto mRNA by a miR-15b mimetic, when introduced in glioma cells. Interestingly, the overexpression of Cripto and the miR-15b mimetic circumvented the inhibitory role of miR-15b on Cripto and the subsequent cell proliferation, invasion, and downregulation of MMP-2 and MMP-9 [80]. This molecular mechanism of direct Cripto control was also previously identified in lung cancer [79]. Cripto expression was detected in 40–50% of human melanomas and melanoma cell lines. The cellular effects of Cripto overexpression included increased cell proliferation and invasion, which were mediated via both Src- and ALK4-dependent signaling pathways. Interference with Cripto mRNA levels directly by siRNA, or via the inhibition of c-Src with Saracatinib, or ALK4 kinase activity via a small molecule inhibitor, abrogated the tumor-promoting effects of Cripto [88]. A high Cripto level was found in 55% of oral squamous carcinoma (OSC) compared to the normal mucosa tissues and correlated with epithelial dysplasia and a poorly differentiated histological grade and proliferation fraction in human tissues. Exogenous Cripto accelerated tumorigenic properties, such as the proliferation and invasion of OSC cell lines [89].

In hepatocellular carcinoma (HCC), it was shown that Cripto promotes stemness by stabilizing Dishevelled-3 and activating the Wnt/β-catenin signaling cascade. The knock down of Cripto also reduced the activity of components of the Wnt/β-catenin pathway, such as β-catenin, AXIN2, and C-MYC [90]. The invasion potential of HCC cells has been shown to be regulated by NANOG, which increased the expression of Nodal and Cripto to promote SMAD3 phosphorylation and SNAIL expression and EMT progression [91]. Furthermore, higher Cripto protein expression was detected in tumor tissues compared to their adjacent non-tumor tissues [5]. Cripto expression correlated with a higher histological grade and disease stage (classification of malignant tumors (TNM) and Barcelona Clinic Liver Cancer (BCLC) staging system) for both early and late recurrence [92]. Cripto expression was also negatively correlated with overall survival [92]. 

Cripto expression and the serum liver marker α-fetoprotein showed significant predictive values when used in combination and may be used as clinical biomarkers for survival risk patient classification and time to recurrence after hepatectomy, for which diagnostic tools are currently lacking in the field of HCC [92].

Cripto has also been strongly linked to human breast cancer [3,86]. In fact, one of the first studies to demonstrate the in vivo oncogenic transformation abilities of Cripto, exerted by induction of the EMT phenotype, involved transgenic models of mouse mammary gland carcinoma [93]. 

Given the high metastasis relapse rate of breast cancer patients, eliciting an immune response against antigens found in cells with a high self-renewal capacity and metastatic potential is critical. Cripto is specifically expressed in cancer cells with stem cell properties, so the effect of an anti-Cripto immune response was tested in xenograft models [94]. An anti-Cripto vaccine effectively reduced the primary and lung metastatic tumor burden in the orthotopic 4T1 mouse BrCa xenograft. In a more clinically relevant spontaneous BrCa model (BALBc NeuT), a Cripto vaccination resulted in a decreased number of lung metastasis foci, but had no effect on the primary tumor, which had low Cripto expression. A prophylactic effect of a Cripto vaccine was observed upon xenografting Cripto expressing tumor cancer stem cell (CSC) spheres [94]. 

Castro et al. also investigated in vivo Cripto targeting and its potential therapeutic implication. In the aggressive triple negative breast cancer type, Cripto was found to be expressed, specifically in the mesenchymal areas of primary tumors from a spontaneous mouse model (JygMC(A)). The injection of Cripto CRISP9-knockout JygMC(A) cells into the mammary pad led to smaller in situ tumor lesions and reduced the lung metastasis foci [95]. Similarly, an anti-Cripto vaccination elicited a strong cytotoxic T cell response and prevented lung metastases in a preclinical model of metastatic melanoma [96]. Therefore, aberrant levels of Cripto contribute to tumor growth and metastasis. Moreover, in lung adenocarcinoma (stage I–III LAC), high Cripto expression is linked to significantly poorer progression free survival and overall survival, and shows high predictability, particularly when combined with serum carcinoembryonic antigen (CEA) levels [97]. An innate tumor resistance to epidermal growth factor receptor (EGFR) small molecule inhibitors was found in 10% of non-small cell and lung carcinoma patients and the Cripto expression was higher in the cases exhibiting resistance compared to therapy responders [97,98]. 

The therapy resistance and high oncogenicity associated with Cripto are potentially due to its roles in promoting maintenance of the stem cell phenotype in normal stem cells and CSCs/tumor-initiating cells [1]. As demonstrated in human embryonal teratocarcinoma (EC) cells, high Cripto-expressing EC cells had a high tumorigenic potential in vivo and in vitro, compared to the Cripto-low counterpart subpopulation [99]. The upregulation of Cripto in these specific cell populations was due to direct regulation by stem cell factors Nanog and Oct4, as well as promoter methylation changes [99]. Therapeutic agents that inhibit CSC proliferation in preclinical breast cancer models have been shown to inhibit specific Cripto-dependent signaling cascades in CSCs [100]. Pretreatment with a sulforaphane compound had in vivo tumor inhibitory effects, which were mediated through reduced Cripto and other stem cell markers, ALDH1A1, Nanog, and homologue CRIPTO-3 expression in triple negative breast cancer [100]. Similarly, in prostate cancer (PCa), Cripto expression was shown to be co-expressed with Oct4 in stem cell subpopulations of PC-3 cell lines [101]. Cripto and GRP78 were enriched in the metastatic aldehyde dehydrogenase (ALDH)-high CSC subpopulation of human bone metastasis cell line PC-3M-Pro4, compared to a non-metastatic ALDH-low subpopulation, and were highly expressed in human bone metastases tissues. In turn, targeting Cripto expression led to a reduction of the ALDH-high CSC population and inhibited bone metastases in a preclinical mouse model [2].

In esophageal squamous cell carcinoma (ESCC), high levels of Cripto have been correlated with a poor prognosis, high invasiveness, and metastatic capabilities. Cripto expression overlaps with ALDH1A1 in ESCC cell lines and specimens. Furthermore, two distinct subpopulations, CRIPTO^high^ and CRIPTO^low^, were isolated from the cell lines, showing that CRIPTO^high^ cells expressed higher levels of Oct4, Nanog, and Sox2 compared to CRIPTO^low^ cells [102]. 

The Cripto binding and signaling partner Nodal has also been investigated in human cancers. In glioblastoma, high Cripto levels did not correlate with high Nodal expression, suggesting that Cripto exerts tumorigenic effects independent from Nodal [87]. However, Nodal reactivation seems to be associated with highly invasive breast carcinomas [103], and an independent study showed that Cripto expression patterns were similar to those of Nodal in high grade and poor prognosis cases [104]. Like Cripto, Nodal is an hESC-associated factor and has been shown to promote breast cancer growth and aggressiveness, and the human breast cancer cell lines MDA-MB-231, MDA-MB-468, and Hs578t have each been shown to present elevated Nodal expression [105]. Nodal is also expressed at low levels in normal tissues, and an increasing Nodal expression coincides with progressive disease in melanoma malignancies [106]. Cripto and Nodal reactivation in normal/benign tissues is an early deterministic factor for carcinoma progression, as investigated in benign melanocytic nevi. Patient follow-up indicated that cases with metastatic melanoma had previously exhibited high Cripto, Nodal, and Notch4 (inducer of Nodal) expression [107]. Interestingly, in individual non-malignant cases, there was no direct correlation between the levels of Nodal and Cripto staining, although in advanced melanoma and metastasis, high expression levels of both proteins were detected, meaning that there may be a threshold level of expression of either Cripto or Nodal, above which the activation of both is synergistically increasing [107]. Nodal inhibition by a targeting antibody in breast cancer cell lines inhibited the proliferation and colony forming ability in vitro [103]. The tumor-promoting activity of Nodal may be enhanced by the lack of expression of its inhibitor Lefty, which is expressed and tightly regulates Nodal activity during embryogenesis, but is absent in cancer cells [108]. 

Therapeutic agents that can prevent Nodal/Cripto interaction and downstream signaling, such as neutralizing Nodal antibodies and ligand traps such as Lefty or Cerberus, have shown promising tumor-inhibiting potential in melanoma, breast, and pancreatic cancers [108,109,110].

### 2.6. Cripto in Experimental Medical Research: Therapeutic and Translational Applications

The almost complete absence of Cripto expression in adult tissues under physiological conditions highlights its potential role as a biomarker for multiple diseases. Genetic imbalance of the TDGF1 locus is per se a risk factor of tumorigenesis, as demonstrated by data from transgenic mouse models with a heterozygous loss of TDGF1. When challenged with a colon-specific carcinogen, Cripto heterozygous mice exhibited a higher incidence of colon cancer compared to Cripto wild-type mice [111]. Polymorphisms and mutations in the TDGF1 locus have been linked to different medical conditions and assessing the TDGF1 genetic status was shown to improve cancer patients’ risk stratification, as well as produce information about potential genetic disorders [2,112,113]. For instance, Cripto promoter methylation was shown to discriminate among different subtypes of testicular germ cell tumors [112]. The potential use of Cripto as a biomarker also showed promising results in breast and colon cancer, with higher levels in the serum of cancer patients compared to healthy individuals [4]. However, its use as a pan-cancer serological marker should be further investigated in order to establish a definite threshold for baseline levels of circulating Cripto. In this regard, a few polymorphisms in the TDGF1 promoter have been linked to a significantly reduced level of circulating Cripto in a genome-wide association study of about 1500 individuals [113]. 

In an approach to identify biomarkers for disease and therapy responses using gene expression arrays, Cripto was one of the markers specifically associated with the metastasis stage, thus discriminating primary intrahepatic cholangiocarcinoma from secondary metastasis [114]. Cripto may also be beneficial as a biomarker to discriminate among diseases that have different treatment approaches. For example, Cripto expression was decreased in pluripotent carcinoma cells following non-ionizing radiation treatment and its reduction was associated with impaired tumorigenicity [115]. Overall, Cripto is commonly associated with advanced or metastatic disease, while the lack of molecular targets or targeted therapies highlights the possibility of Cripto as a pan-cancer prediction biomarker or therapeutic target in combination with treatment schemes. 

Cripto has shown heterogeneous effects in tumorigenesis and tissue regeneration, depending on its expression levels and signaling pathway activation status. Advances in non-invasive techniques were applied to studies of Cripto as a stem cell factor in cell tissue engineering. The term tissue engineering refers to a set of specific techniques that aim to replace or repair a damaged tissue with the aid of natural or synthetic scaffolds. This type of approach frequently requires the use of embryonic and/or pluripotent adult stem cells (ESC and iPSC, respectively). Although experimental research presents significant barriers hindering the clinical use of hESCs and iPSC, including ethical issues, immunorejection, and the tumorigenic or teratogenic potential of these cells, the key role of Cripto as a regulator of embryonic and adult stem cells [116,117,118] has led to sustained interest in its investigation in the field of tissue engineering. Studies on protein–protein interactions, early induced pluripotent stem cell (iPCS) reprogramming, and embryonic development have shed light on the regulatory networks of Cripto [119,120]. Cripto was identified as a key player in hematopoietic stem cell (HSC) biology [117,121]. A specific subpopulation of HSC was identified based on its high levels of cell surface GRP78 expression and shown to reside in the endosteal area of the bone. These HSC GRP78+ cells showed hypoxic features and lower mitochondrial activity, and their HSC potential in vitro was strictly dependent on Cripto-induced glycolytic activity. Interestingly, HIF1A1 KO mice displayed a reduced number of HSC GRP78+ cells, together with lower levels of Cripto expression, suggesting Cripto/GRP78 signaling as a mediator of HIF1A expression [117]. Cripto and GRP78 were also identified as regulators of stem cell behavior in isolated primary fetal and adult mammary epithelial cells [116]. Cripto specifically maintains the stem cell phenotype in these cultures and favors colonies with an enhanced mammary gland reconstitution capacity, while GRP78 deletion from adult mammary epithelial cells blocks their mammary gland reconstitution potential [116].

In 2010, a Cripto blocking peptide (Cripto-BP) was identified that prevents Cripto /Alk-4 receptor interaction and interferes with Cripto signaling [122]. This Cripto-specific blocking tool, which mimics the effect of the genetic ablation of Cripto, improved neural induction and dopaminergic differentiation in ESC lines and enhanced the functional integration of mouse ESC [122]. iPSC-mesenchymal stem cells (MSCs) were proven to lack stemness markers (including Cripto), while showing adequate differentiation potential, osteogenic and chondrogenic properties, and MSC marker expression compared to bone-marrow-derived MSC (BM-MSCs) [123]. 

Furthermore, a study focusing on the development of antibodies targeting Cripto with a neutralizing effect in vitro was published [124]. The research reported the generation and characterization of murine monoclonal antibodies raised against the synthetic folded CFC domain of human Cripto. The authors focused on antibodies targeting the “hot spots” of the CFC domain crucial for Activin Type I receptor (ALK4) and GRP78 interaction. Among the antibodies tested, the 1B4 antibody was able to bind the membrane anchored and soluble forms of native Cripto protein in a panel of human cancer cells and also interfered with downstream Cripto-dependent signaling [124]. In many studies, Cripto has been immunolocalized using anti-Cripto antibodies, which is an approach that was systematically reviewed by Gudbergsson et al. [125]. 

## 3. Materials and Methods 

### 3.1. Search Strategy

To identify the complete collection of Cripto-related scientific literature, the WoS Core Collection database was used to list all of the scientific documents with the search criteria “Cripto OR Tdgf1”, including the title, abstract, and keyword fields, during the period 1900–2020, up to 9 April 2020. We retrieved the scientific literature within the Science Citation Index Expanded (SSCI-EXPANDED) and Social Sciences Citation Index (SSCI) with no language restriction.

### 3.2. Citation and Publication Data

A total number of 584 publications were collected and analyzed by diverse fields, such as authors (AU), author’s country (AU_CO), publication source (SO), publication year (PY), author’s keywords (DE), keywords associated with the WoS database (ID), and citation data (CR). The authors were determined as the list of authors on the respective publication. The country of origin was defined as the corresponding first author’s country. The publication year, author’s keywords, keywords associated with the WoS database, and citation data were established as per indexing on the WoS database [126].

### 3.3. Data Analysis

The data obtained from every category were processed using the bibliometrix R package [24] and processed with R version 3.6.3 [127] in R Studio software version 1.2.5033 [128], in order to perform descriptive analyses of the bibliographic data frame. 

The Authors per article Index was calculated as the ratio between the total number of articles and the total number of authors. The Co-Authors per Articles Index was calculated as the average number of co-authors per article. The Collaboration Index (CI) was calculated as the Total Authors of Multi-Authored Articles/Total Multi-Authored Articles [129,130]. 

Authors’ dominance ranking was calculated as proposed by Kumar and Kumar, 2008 [131]. Manuscript coupling, reference co-citation, and keyword co-occurrence were calculated using the function “biblionetwork” within the bibliometrix R package [24]. The function “citations” was used to define cited references (CR) and to generate a frequency table of the most cited references or the most cited first authors (among the references) [24].

To map the conceptual structure of a framework, we used the word co-occurrences in a bibliographic collection to generate a conceptual structure map, a topic dendrogram, a factorial map of the documents with the highest contribution, and a factorial map of the most cited documents for each category. The keyword co-occurrence network, considered the outline principal research method of researchers, can be used to understand the knowledge components and knowledge structure of a scientific/technical field by examining the links between keywords in the literature [132]. The conceptual structure of the network was estimated using the function “ConceptualStructure” within the bibliometrix R package [24].

In conclusion, wordclouds of the most frequent keywords in the surveyed literature were generated using the “wordcloud” R package [133].

## 4. Conclusions

In this review, we compiled, for the first time, a bibliometric analysis of studies focusing on Cripto signaling and used this approach as a leading strategy to review clusters of research fields. The oncofetal protein Cripto has been shown to play important roles in regulating stem cell differentiation and embryogenesis, and ultimately to be involved in tumorigenesis. During embryogenesis, Cripto has been shown to have a dual role both in Nodal-dependent and -independent pathways. Cripto mutations or deletion show defects in development, especially in AP pattern formation and cardiac development in different animal models. Moreover, Cripto and its signaling partner GRP78 present a complex network of interactions. They have been defined as modulators of TGF-β, Src, MAPK, and PI3K pathways and as regulators of signaling pathways such as those of Wnt and Notch. 

We have shown that a high percentage of studies related to Cripto are focused on its oncogenic role. In adult physiological conditions, Cripto expression levels have been shown to be negligible, whereas, in human tumors, elevated Cripto levels promote EMT, angiogenesis, augment the invasion capability, and increase cell proliferation and migration in both in vitro and in vivo models. One of the first studies to demonstrate the in vivo oncogenic features of Cripto was conducted in transgenic models of mouse mammary gland carcinoma, proposing a strong link between Cripto and human breast cancer. Furthermore, a high expression of Cripto is associated with a poorer prognosis and worse overall survival in a variety of cancers, such as glioblastoma, glioma, melanomas, and lung adenocarcinoma. Cripto has also been shown to interact with the Wnt/β-catenin signaling cascade, promoting stemness potential in hepatocellular cancer.

Interestingly, researchers have also started to explore the therapeutic potential of Cripto and have proposed an anti-Cripto vaccination and therapies eliciting an anti-Cripto immune response.

As highlighted in this review, Cripto carries a great therapeutic and diagnostic potential, but this potential does not come without challenges. The complex network of signaling orbiting around Cripto that has started to unravel in the last 10 years appears to be only the tip of the iceberg. Specific studies aiming to unravel the molecular signaling of this protein are still needed, as well as lineage tracing studies that could give a more comprehensive view of the pattern of the expression of Cripto and shed light on its possible role in adult development.

Our bibliometric analysis provides a comprehensive overview of the dynamic research related to this oncofetal protein. Cripto accounts for a considerable number of publications, mostly in oncology, cell biology and biochemistry, and molecular biology categories within the contemporary scientific literature. We have discussed multiple aspects of Cripto research and potential areas of applicability to cancer therapy and regenerative medicine. We have specifically highlighted the research conducted in the last 10 years, which is when the majority of publications and studies were conducted. 

The use of only one database (WoS) for the research may be a limitation. In fact, a number of alternate databases that calculate citation metrics are available (SCOPUS, Cochrane Database of Systematic Reviews, and PubMed). However, WoS covers the oldest publications compared to SCOPUS, which is limited to recent articles (citation analyses are only available for articles published after 1996). Furthermore, WoS provides more detailed citation analyses and graphics compared to SCOPUS [134] and Pubmed, which do not provide citation analysis. In our analysis, some studies may have been unintentionally excluded due to keyword selection and limited indexing.

Despite these limitations, the use of a bibliometric approach to retrieve comprehensive publication records is a promising strategy for summarizing a research topic in a systematic way. In terms of the ability to analyze metadata related to research studies, it is important to have a data-driven approach, even during a review process. In conclusion, we believe that the summary of the research findings, combined with conceptual, social, and intellectual networks, highlights the relevance of including multiple categories when reviewing scientific literature. Finally, this approach is aimed at helping Cripto researchers identify key areas that remain to be investigated, and the most suitable potential collaborators with whom to conduct their studies. Ultimately, the scientific community would benefit from such a targeted approach because it offers a facilitated and accurate examination of the scientific literature available for a specific field of research. 

## Supplementary Materials

The following are available online at https://www.mdpi.com/2072-6694/12/6/1480/s1: Figure S1: Wordcloud of keywords.

## References

[1] Klauzinska M., Castro N.P., Rangel M.C., Spike B.T., Gray P.C., Bertolette D., Cuttitta F., Salomon D. (2014). The multifaceted role of the embryonic gene Cripto-1 in cancer, stem cells and epithelial-mesenchymal transition. Semin. Cancer Biol..

[2] Zoni E., Chen L., Karkampouna S., Granchi Z., Verhoef E.I., La Manna F., Kelber J., Pelger R.C.M., Henry M.D., Snaar-Jagalska E. (2017). CRIPTO and its signaling partner GRP78 drive the metastatic phenotype in human osteotropic prostate cancer. Oncogene.

[3] Qi C.F., Liscia D.S., Normanno N., Merlo G., Johnson G.R., Gullick W.J., Ciardiello F., Saeki T., Brandt R., Kim N. (1994). Expression of transforming growth factor alpha, amphiregulin and cripto-1 in human breast carcinomas. Br. J. Cancer.

[4] Bianco C., Strizzi L., Mancino M., Rehman A., Hamada S., Watanabe K., De Luca A., Jones B., Balogh G., Russo J. (2006). Identification of cripto-1 as a novel serologic marker for breast and colon cancer. Clin. Cancer Res..

[5] Karkampouna S., van der Helm D., Gray P.C., Chen L., Klima I., Grosjean J., Burgmans M.C., Farina-Sarasqueta A., Snaar-Jagalska E.B., Stroka D.M. (2018). CRIPTO promotes an aggressive tumour phenotype and resistance to treatment in hepatocellular carcinoma. J. Pathol..

[6] Pilgaard L., Mortensen J.H., Henriksen M., Olesen P., Sørensen P., Laursen R., Vyberg M., Agger R., Zachar V., Moos T. (2014). Cripto-1 expression in glioblastoma multiforme. Brain Pathol. Zurich Switz..

[7] Xu C.H., Wang Y., Qian L.H., Yu L.K., Zhang X.W., Wang Q.B. (2017). Serum Cripto-1 is a novel biomarker for non-small cell lung cancer diagnosis and prognosis. Clin. Respir. J..

[8] Gray P.C., Harrison C.A., Vale W. (2003). Cripto forms a complex with activin and type II activin receptors and can block activin signaling. Proc. Natl. Acad. Sci. USA.

[9] Adkins H.B., Bianco C., Schiffer S.G., Rayhorn P., Zafari M., Cheung A.E., Orozco O., Olson D., Luca A.D., Chen L.L. (2003). Antibody blockade of the Cripto CFC domain suppresses tumor cell growth in vivo. J. Clin. Investig..

[10] Gray P.C., Shani G., Aung K., Kelber J., Vale W. (2006). Cripto Binds Transforming Growth Factor β (TGF-β) and Inhibits TGF-β Signaling. Mol. Cell. Biol..

[11] Kelber J.A., Panopoulos A.D., Shani G., Booker E.C., Belmonte J.C., Vale W.W., Gray P.C. (2009). Blockade of Cripto binding to cell surface GRP78 inhibits oncogenic Cripto signaling via MAPK/PI3K and Smad2/3 pathways. Oncogene.

[12] Gray P.C., Vale W. (2012). Cripto/GRP78 modulation of the TGF-β pathway in development and oncogenesis. FEBS Lett..

[13] Shen M.M. (2007). Nodal signaling: developmental roles and regulation. Dev. Camb. Engl..

[14] Cheng S.K., Olale F., Bennett J.T., Brivanlou A.H., Schier A.F. (2003). EGF-CFC proteins are essential coreceptors for the TGF-beta signals Vg1 and GDF1. Genes Dev..

[15] Chen C., Ware S.M., Sato A., Houston-Hawkins D.E., Habas R., Matzuk M.M., Shen M.M., Brown C.W. (2006). The Vg1-related protein Gdf3 acts in a Nodal signaling pathway in the pre-gastrulation mouse embryo. Dev. Camb. Engl..

[16] Shani G., Fischer W.H., Justice N.J., Kelber J.A., Vale W., Gray P.C. (2008). GRP78 and Cripto form a complex at the cell surface and collaborate to inhibit transforming growth factor beta signaling and enhance cell growth. Mol. Cell. Biol..

[17] Nagaoka T., Karasawa H., Turbyville T., Rangel M.-C., Castro N.P., Gonzales M., Baker A., Seno M., Lockett S., Greer Y.E. (2013). Cripto-1 enhances the canonical Wnt/β-catenin signaling pathway by binding to LRP5 and LRP6 co-receptors. Cell. Signal..

[18] Gao Y., Ge L., Shi S., Sun Y., Liu M., Wang B., Shang Y., Wu J., Tian J. (2019). Global trends and future prospects of e-waste research: a bibliometric analysis. Environ. Sci. Pollut. Res. Int..

[19] He L., Fang H., Chen C., Wu Y., Wang Y., Ge H., Wang L., Wan Y., He H. (2020). Metastatic castration-resistant prostate cancer: Academic insights and perspectives through bibliometric analysis. Medicine (Baltimore).

[20] Lou J., Tian S.-J., Niu S.-M., Kang X.-Q., Lian H.-X., Zhang L.-X., Zhang J.-J. (2020). Coronavirus disease 2019: A bibliometric analysis and review. Eur. Rev. Med. Pharmacol. Sci..

[21] Van Eck N.J., Waltman L. (2017). Citation-based clustering of publications using CitNetExplorer and VOSviewer. Scientometrics.

[22] Synnestvedt M.B., Chen C., Holmes J.H. (2005). CiteSpace II: visualization and knowledge discovery in bibliographic databases. AMIA Annu. Symp. Proc. AMIA Symp..

[23] Van Eck N.J., Waltman L. (2010). Software survey: VOSviewer, a computer program for bibliometric mapping. Scientometrics.

[24] Aria M., Cuccurullo C. (2017). bibliometrix: An R-tool for comprehensive science mapping analysis. J. Informetr..

[25] Ciccodicola A., Dono R., Obici S., Simeone A., Zollo M., Persico M.G. (1989). Molecular characterization of a gene of the “EGF family” expressed in undifferentiated human NTERA2 teratocarcinoma cells. EMBO J..

[26] Ciardiello F., Dono R., Kim N., Persico M.G., Salomon D.S. (1991). Expression of cripto, a novel gene of the epidermal growth factor gene family, leads to in vitro transformation of a normal mouse mammary epithelial cell line. Cancer Res..

[27] Brennan J., Lu C.C., Norris D.P., Rodriguez T.A., Beddington R.S., Robertson E.J. (2001). Nodal signalling in the epiblast patterns the early mouse embryo. Nature.

[28] Guzman-Ayala M., Ben-Haim N., Beck S., Constam D.B. (2004). Nodal protein processing and fibroblast growth factor 4 synergize to maintain a trophoblast stem cell microenvironment. Proc. Natl. Acad. Sci. USA.

[29] Mesnard D. (2006). Nodal specifies embryonic visceral endoderm and sustains pluripotent cells in the epiblast before overt axial patterning. Development.

[30] Blakeley P., Fogarty N.M.E., del Valle I., Wamaitha S.E., Hu T.X., Elder K., Snell P., Christie L., Robson P., Niakan K.K. (2015). Defining the three cell lineages of the human blastocyst by single-cell RNA-seq. Dev. Camb. Engl..

[31] Schier A.F. (2003). Nodal signaling in vertebrate development. Annu. Rev. Cell Dev. Biol..

[32] Schier A.F., Shen M.M. (2000). Nodal signalling in vertebrate development. Nature.

[33] Whitman M. (2001). Nodal signaling in early vertebrate embryos: themes and variations. Dev. Cell.

[34] Fiorenzano A., Pascale E., D’Aniello C., Acampora D., Bassalert C., Russo F., Andolfi G., Biffoni M., Francescangeli F., Zeuner A. (2016). Cripto is essential to capture mouse epiblast stem cell and human embryonic stem cell pluripotency. Nat. Commun..

[35] Hall V.J., Hyttel P. (2014). Breaking down pluripotency in the porcine embryo reveals both a premature and reticent stem cell state in the inner cell mass and unique expression profiles of the naive and primed stem cell states. Stem Cells Dev..

[36] Van Leeuwen J., Berg D.K., Pfeffer P.L. (2015). Morphological and Gene Expression Changes in Cattle Embryos from Hatched Blastocyst to Early Gastrulation Stages after Transfer of In Vitro Produced Embryos. PLoS ONE.

[37] Bianco C., Cotten C., Lonardo E., Strizzi L., Baraty C., Mancino M., Gonzales M., Watanabe K., Nagaoka T., Berry C. (2009). Cripto-1 is required for hypoxia to induce cardiac differentiation of mouse embryonic stem cells. Am. J. Pathol..

[38] Kruithof-de Julio M., Alvarez M.J., Galli A., Chu J., Price S.M., Califano A., Shen M.M. (2011). Regulation of extra-embryonic endoderm stem cell differentiation by Nodal and Cripto signaling. Dev. Camb. Engl..

[39] Takaoka K., Nishimura H., Hamada H. (2017). Both Nodal signalling and stochasticity select for prospective distal visceral endoderm in mouse embryos. Nat. Commun..

[40] Chu J., Shen M.M. (2010). Functional redundancy of EGF-CFC genes in epiblast and extraembryonic patterning during early mouse embryogenesis. Dev. Biol..

[41] Kimura C., Shen M.M., Takeda N., Aizawa S., Matsuo I. (2001). Complementary functions of Otx2 and Cripto in initial patterning of mouse epiblast. Dev. Biol..

[42] Conlon F.L., Lyons K.M., Takaesu N., Barth K.S., Kispert A., Herrmann B., Robertson E.J. (1994). A primary requirement for nodal in the formation and maintenance of the primitive streak in the mouse. Dev. Camb. Engl..

[43] Liguori G.L., Echevarría D., Improta R., Signore M., Adamson E., Martínez S., Persico M.G. (2003). Anterior neural plate regionalization in cripto null mutant mouse embryos in the absence of node and primitive streak. Dev. Biol..

[44] Perea-Gomez A., Vella F.D.J., Shawlot W., Oulad-Abdelghani M., Chazaud C., Meno C., Pfister V., Chen L., Robertson E., Hamada H. (2002). Nodal antagonists in the anterior visceral endoderm prevent the formation of multiple primitive streaks. Dev. Cell.

[45] Rivera-Pérez J.A., Magnuson T. (2005). Primitive streak formation in mice is preceded by localized activation of Brachyury and Wnt3. Dev. Biol..

[46] Ding J., Yang L., Yan Y.T., Chen A., Desai N., Wynshaw-Boris A., Shen M.M. (1998). Cripto is required for correct orientation of the anterior-posterior axis in the mouse embryo. Nature.

[47] Varlet I., Collignon J., Norris D.P., Robertson E.J. (1997). Nodal signaling and axis formation in the mouse. Cold Spring Harb. Symp. Quant. Biol..

[48] Jin J.-Z., Zhu Y., Warner D., Ding J. (2016). Analysis of extraembryonic mesodermal structure formation in the absence of morphological primitive streak. Dev. Growth Differ..

[49] Roessler E., Ouspenskaia M.V., Karkera J.D., Vélez J.I., Kantipong A., Lacbawan F., Bowers P., Belmont J.W., Towbin J.A., Goldmuntz E. (2008). Reduced NODAL signaling strength via mutation of several pathway members including FOXH1 is linked to human heart defects and holoprosencephaly. Am. J. Hum. Genet..

[50] Lin X., Zhao W., Jia J., Lin T., Xiao G., Wang S., Lin X., Liu Y., Chen L., Qin Y. (2016). Ectopic expression of Cripto-1 in transgenic mouse embryos causes hemorrhages, fatal cardiac defects and embryonic lethality. Sci. Rep..

[51] Osório L., Wu X., Wang L., Jiang Z., Neideck C., Sheng G., Zhou Z. (2019). ISM1 regulates NODAL signaling and asymmetric organ morphogenesis during development. J. Cell Biol..

[52] Lee G.-H., Fujita M., Takaoka K., Murakami Y., Fujihara Y., Kanzawa N., Murakami K.-I., Kajikawa E., Takada Y., Saito K. (2016). A GPI processing phospholipase A2, PGAP6, modulates Nodal signaling in embryos by shedding CRIPTO. J. Cell Biol..

[53] Farina A., D’Aniello C., Severino V., Hochstrasser D.F., Parente A., Minchiotti G., Chambery A. (2011). Temporal proteomic profiling of embryonic stem cell secretome during cardiac and neural differentiation. Proteomics.

[54] Feldman B., Gates M.A., Egan E.S., Dougan S.T., Rennebeck G., Sirotkin H.I., Schier A.F., Talbot W.S. (1998). Zebrafish organizer development and germ-layer formation require nodal-related signals. Nature.

[55] Schier A.F., Talbot W.S. (2001). Nodal signaling and the zebrafish organizer. Int. J. Dev. Biol..

[56] Shah S.B., Skromne I., Hume C.R., Kessler D.S., Lee K.J., Stern C.D., Dodd J. (1997). Misexpression of chick Vg1 in the marginal zone induces primitive streak formation. Dev. Camb. Engl..

[57] Sirotkin H.I., Dougan S.T., Schier A.F., Talbot W.S. (2000). bozozok and squint act in parallel to specify dorsal mesoderm and anterior neuroectoderm in zebrafish. Dev. Camb. Engl..

[58] Levin M., Johnson R.L., Stern C.D., Kuehn M., Tabin C. (1995). A molecular pathway determining left-right asymmetry in chick embryogenesis. Cell.

[59] Whitman M., Mercola M. (2001). TGF-beta superfamily signaling and left-right asymmetry. Sci. STKE Signal Transduct. Knowl. Environ..

[60] Fleming B.M., Yelin R., James R.G., Schultheiss T.M. (2013). A role for Vg1/Nodal signaling in specification of the intermediate mesoderm. Dev. Camb. Engl..

[61] Porokh V., Vaňhara P., Bárta T., Jurečková L., Bohačiaková D., Pospíšilová V., Mináriková D., Holubcová Z., Pelková V., Souček K. (2018). Soluble Cripto-1 Induces Accumulation of Supernumerary Centrosomes and Formation of Aberrant Mitoses in Human Embryonic Stem Cells. Stem Cells Dev..

[62] Hamdi M., Sánchez-Calabuig M.J., Rodríguez-Alonso B., Bagés Arnal S., Roussi K., Sturmey R., Gutiérrez-Adán A., Lonergan P., Rizos D. (2019). Gene expression and metabolic response of bovine oviduct epithelial cells to the early embryo. Reprod. Camb. Engl..

[63] Clemente M., Lopez-Vidriero I., O’Gaora P., Mehta J.P., Forde N., Gutierrez-Adan A., Lonergan P., Rizos D. (2011). Transcriptome changes at the initiation of elongation in the bovine conceptus. Biol. Reprod..

[64] Papageorgiou I., Nicholls P.K., Wang F., Lackmann M., Makanji Y., Salamonsen L.A., Robertson D.M., Harrison C.A. (2009). Expression of nodal signalling components in cycling human endometrium and in endometrial cancer. Reprod. Biol. Endocrinol. RBE.

[65] Carrarelli P., Yen C.-F., Arcuri F., Funghi L., Tosti C., Wang T.-H., Huang J.S., Petraglia F. (2015). Myostatin, follistatin and activin type II receptors are highly expressed in adenomyosis. Fertil. Steril..

[66] Cruz C.D., Del Puerto H.L., Rocha A.L.L., Cavallo I.K., Clarizia A.D., Petraglia F., Reis F.M. (2015). Expression of Nodal, Cripto, SMAD3, phosphorylated SMAD3, and SMAD4 in the proliferative endometrium of women with endometriosis. Reprod. Sci. Thousand Oaks Calif.

[67] Banz C., Ungethuem U., Kuban R.-J., Diedrich K., Lengyel E., Hornung D. (2010). The molecular signature of endometriosis-associated endometrioid ovarian cancer differs significantly from endometriosis-independent endometrioid ovarian cancer. Fertil. Steril..

[68] Torricelli M., Voltolini C., Novembri R., Bocchi C., Di Tommaso M., Severi F.M., Petraglia F. (2012). Activin A and its regulatory molecules in placenta and fetal membranes of women with preterm premature rupture of the membranes associated with acute chorioamnionitis. Am. J. Reprod. Immunol..

[69] Bandeira C.L., Urban Borbely A., Pulcineli Vieira Francisco R., Schultz R., Zugaib M., Bevilacqua E. (2014). Tumorigenic factor CRIPTO-1 is immunolocalized in extravillous cytotrophoblast in placenta creta. BioMed Res. Int..

[70] Kelber J.A., Shani G., Booker E.C., Vale W.W., Gray P.C. (2008). Cripto is a noncompetitive activin antagonist that forms analogous signaling complexes with activin and nodal. J. Biol. Chem..

[71] Ciarmela P., Bloise E., Gray P.C., Carrarelli P., Islam M.S., De Pascalis F., Severi F.M., Vale W., Castellucci M., Petraglia F. (2011). Activin-A and Myostatin Response and Steroid Regulation in Human Myometrium: Disruption of Their Signalling in Uterine Fibroid. J. Clin. Endocrinol. Metab..

[72] Kouznetsova V.L., Hu H., Teigen K., Zanetti M., Tsigelny I.F. (2018). Cripto stabilizes GRP78 on the cell membrane. Protein Sci. Publ. Protein Soc..

[73] Hamada S., Watanabe K., Hirota M., Bianco C., Strizzi L., Mancino M., Gonzales M., Salomon D.S. (2007). beta-Catenin/TCF/LEF regulate expression of the short form human Cripto-1. Biochem. Biophys. Res. Commun..

[74] Mancino M., Strizzi L., Wechselberger C., Watanabe K., Gonzales M., Hamada S., Normanno N., Salomon D.S., Bianco C. (2008). Regulation of human Cripto-1 gene expression by TGF-beta1 and BMP-4 in embryonal and colon cancer cells. J. Cell. Physiol..

[75] Pilli V.S., Gupta K., Kotha B.P., Aradhyam G.K. (2015). Snail-mediated Cripto-1 repression regulates the cell cycle and epithelial-mesenchymal transition-related gene expression. FEBS Lett..

[76] Bianco C., Castro N.P., Baraty C., Rollman K., Held N., Rangel M.C., Karasawa H., Gonzales M., Strizzi L., Salomon D.S. (2013). Regulation of human Cripto-1 expression by nuclear receptors and DNA promoter methylation in human embryonal and breast cancer cells. J. Cell. Physiol..

[77] Su R., Cao S., Ma J., Liu Y., Liu X., Zheng J., Chen J., Liu L., Cai H., Li Z. (2017). Knockdown of SOX2OT inhibits the malignant biological behaviors of glioblastoma stem cells via up-regulating the expression of miR-194-5p and miR-122. Mol. Cancer.

[78] Loying P., Manhas J., Sen S., Bose B. (2015). Autoregulation and heterogeneity in expression of human Cripto-1. PLoS ONE.

[79] Chen F., Hou S., Fan H., Liu Y. (2014). MiR-15a-16 represses Cripto and inhibits NSCLC cell progression. Mol. Cell. Biochem..

[80] Sun G., Yan S.-S., Shi L., Wan Z.-Q., Jiang N., Fu L.-S., Li M., Guo J. (2016). MicroRNA-15b suppresses the growth and invasion of glioma cells through targeted inhibition of cripto-1 expression. Mol. Med. Rep..

[81] Lee S.I., Jeon M., Kim J.S., Jeon I.-S., Byun S.J. (2015). The gga-let-7 family post-transcriptionally regulates *TGFBR1* and *LIN28B* during the differentiation process in early chick development: R EGULATORY R OLE
OF gga-let-7 F AMILY IN E ARLY C HICK D EVELOPMENT. Mol. Reprod. Dev..

[82] Hoover M., Runa F., Booker E., Diedrich J.K., Duell E., Williams B., Arellano-Garcia C., Uhlendorf T., La Kim S., Fischer W. (2019). Identification of myosin II as a cripto binding protein and regulator of cripto function in stem cells and tissue regeneration. Biochem. Biophys. Res. Commun..

[83] Guardiola O., Lafuste P., Brunelli S., Iaconis S., Touvier T., Mourikis P., De Bock K., Lonardo E., Andolfi G., Bouché A. (2012). Cripto regulates skeletal muscle regeneration and modulates satellite cell determination by antagonizing myostatin. Proc. Natl. Acad. Sci. USA.

[84] Kemaladewi D.U., de Gorter D.J.J., Aartsma-Rus A., van Ommen G.-J., ten Dijke P., ’t Hoen P.A.C., Hoogaars W.M. (2012). Cell-type specific regulation of myostatin signaling. FASEB J..

[85] Iavarone F., Guardiola O., Scagliola A., Andolfi G., Esposito F., Serrano A., Perdiguero E., Brunelli S., Muñoz-Cánoves P., Minchiotti G. (2020). Cripto shapes macrophage plasticity and restricts EndMT in injured and diseased skeletal muscle. EMBO Rep..

[86] De Castro N.P., Rangel M.C., Nagaoka T., Salomon D.S., Bianco C. (2010). Cripto-1: an embryonic gene that promotes tumorigenesis. Future Oncol. Lond. Engl..

[87] Tysnes B.B., Satran H.A., Mork S.J., Margaryan N.V., Eide G.E., Petersen K., Strizzi L., Hendrix M.J.C. (2013). Age-Dependent Association between Protein Expression of the Embryonic Stem Cell Marker Cripto-1 and Survival of Glioblastoma Patients. Transl. Oncol..

[88] De Luca A., Lamura L., Strizzi L., Roma C., D’Antonio A., Margaryan N., Pirozzi G., Hsu M.-Y., Botti G., Mari E. (2011). Expression and functional role of CRIPTO-1 in cutaneous melanoma. Br. J. Cancer.

[89] Yoon H.-J., Hong J.-S., Shin W.-J., Lee Y.-J., Hong K.-O., Lee J.-I., Hong S.-P., Hong S.-D. (2011). The role of Cripto-1 in the tumorigenesis and progression of oral squamous cell carcinoma. Oral Oncol..

[90] Lo R.C.-L., Leung C.O.-N., Chan K.K.-S., Ho D.W.-H., Wong C.-M., Lee T.K.-W., Ng I.O.-L. (2018). Cripto-1 contributes to stemness in hepatocellular carcinoma by stabilizing Dishevelled-3 and activating Wnt/β-catenin pathway. Cell Death Differ..

[91] Sun C., Sun L., Jiang K., Gao D.-M., Kang X.-N., Wang C., Zhang S., Huang S., Qin X., Li Y. (2013). NANOG promotes liver cancer cell invasion by inducing epithelial-mesenchymal transition through NODAL/SMAD3 signaling pathway. Int. J. Biochem. Cell Biol..

[92] Wang J.-H., Wei W., Xu J., Guo Z.-X., Xiao C.-Z., Zhang Y.-F., Jian P.-E., Wu X.-L., Shi M., Guo R.-P. (2015). Elevated expression of Cripto-1 correlates with poor prognosis in hepatocellular carcinoma. Oncotarget.

[93] Strizzi L., Bianco C., Normanno N., Seno M., Wechselberger C., Wallace-Jones B., Khan N.I., Hirota M., Sun Y., Sanicola M. (2004). Epithelial mesenchymal transition is a characteristic of hyperplasias and tumors in mammary gland from MMTV-Cripto-1 transgenic mice. J. Cell. Physiol..

[94] Witt K., Ligtenberg M.A., Conti L., Lanzardo S., Ruiu R., Wallmann T., Tufvesson-Stiller H., Chambers B.J., Rolny C., Lladser A. (2018). Cripto-1 Plasmid DNA Vaccination Targets Metastasis and Cancer Stem Cells in Murine Mammary Carcinoma. Cancer Immunol. Res..

[95] Castro N.P., Fedorova-Abrams N.D., Merchant A.S., Rangel M.C., Nagaoka T., Karasawa H., Klauzinska M., Hewitt S.M., Biswas K., Sharan S.K. (2015). Cripto-1 as a novel therapeutic target for triple negative breast cancer. Oncotarget.

[96] Ligtenberg M.A., Witt K., Galvez-Cancino F., Sette A., Lundqvist A., Lladser A., Kiessling R. (2016). Cripto-1 vaccination elicits protective immunity against metastatic melanoma. Oncoimmunology.

[97] Zhang H., Zhang B., Gao L., Zhang L., Zhu K., Cheng R., Wang C. (2017). Clinical significance of cripto-1 expression in lung adenocarcinoma. Oncotarget.

[98] Park K.-S., Raffeld M., Moon Y.W., Xi L., Bianco C., Pham T., Lee L.C., Mitsudomi T., Yatabe Y., Okamoto I. (2014). CRIPTO1 expression in EGFR-mutant NSCLC elicits intrinsic EGFR-inhibitor resistance. J. Clin. Investig..

[99] Watanabe K., Meyer M.J., Strizzi L., Lee J.M., Gonzales M., Bianco C., Nagaoka T., Farid S.S., Margaryan N., Hendrix M.J.C. (2010). Cripto-1 is a cell surface marker for a tumorigenic, undifferentiated subpopulation in human embryonal carcinoma cells. Stem Cells Dayt. Ohio.

[100] Castro N.P., Rangel M.C., Merchant A.S., MacKinnon G., Cuttitta F., Salomon D.S., Kim Y.S. (2019). Sulforaphane Suppresses the Growth of Triple-negative Breast Cancer Stem-like Cells In vitro and In vivo. Cancer Prev. Res. Phila. Pa.

[101] Cocciadiferro L., Miceli V., Kang K.-S., Polito L.M., Trosko J.E., Carruba G. (2009). Profiling cancer stem cells in androgen-responsive and refractory human prostate tumor cell lines. Ann. N. Y. Acad. Sci..

[102] Liu Q., Cui X., Yu X., Bian B.-S.-J., Qian F., Hu X.-G., Ji C.-D., Yang L., Ren Y., Cui W. (2017). Cripto-1 acts as a functional marker of cancer stem-like cells and predicts prognosis of the patients in esophageal squamous cell carcinoma. Mol. Cancer.

[103] Strizzi L., Hardy K.M., Margaryan N.V., Hillman D.W., Seftor E.A., Chen B., Geiger X.J., Thompson E.A., Lingle W.L., Andorfer C.A. (2012). Potential for the embryonic morphogen Nodal as a prognostic and predictive biomarker in breast cancer. Breast Cancer Res. BCR.

[104] Gong Y.P., Yarrow P.M., Carmalt H.L., Kwun S.Y., Kennedy C.W., Lin B.P.C., Xing P.X., Gillett D.J. (2007). Overexpression of Cripto and its prognostic significance in breast cancer: A study with long-term survival. Eur. J. Surg. Oncol..

[105] Quail D.F., Zhang G., Walsh L.A., Siegers G.M., Dieters-Castator D.Z., Findlay S.D., Broughton H., Putman D.M., Hess D.A., Postovit L.-M. (2012). Embryonic morphogen nodal promotes breast cancer growth and progression. PLoS ONE.

[106] Yu L., Harms P.W., Pouryazdanparast P., Kim D.S., Ma L., Fullen D.R. (2010). Expression of the embryonic morphogen Nodal in cutaneous melanocytic lesions. Mod. Pathol..

[107] Strizzi L., Margaryan N.V., Gerami P., Haghighat Z., Harms P.W., Madonna G., Botti G., Ascierto P.A., Hendrix M.J.C. (2016). Translational significance of Nodal, Cripto-1 and Notch4 in adult nevi. Oncol. Lett..

[108] Kalyan A., Carneiro B.A., Chandra S., Kaplan J., Chae Y.K., Matsangou M., Hendrix M.J.C., Giles F. (2017). Nodal Signaling as a Developmental Therapeutics Target in Oncology. Mol. Cancer Ther..

[109] Strizzi L., Hardy K.M., Kirschmann D.A., Ahrlund-Richter L., Hendrix M.J.C. (2012). Nodal expression and detection in cancer: experience and challenges. Cancer Res..

[110] Aykul S., Ni W., Mutatu W., Martinez-Hackert E. (2015). Human Cerberus prevents nodal-receptor binding, inhibits nodal signaling, and suppresses nodal-mediated phenotypes. PLoS ONE.

[111] Giorgio E., Liguoro A., D’Orsi L., Mancinelli S., Barbieri A., Palma G., Arra C., Liguori G.L. (2014). Cripto haploinsufficiency affects in vivo colon tumor development. Int. J. Oncol..

[112] Costa A.L., Moreira-Barbosa C., Lobo J., Vilela-Salgueiro B., Cantante M., Guimarães R., Lopes P., Braga I., Oliveira J., Antunes L. (2018). DNA methylation profiling as a tool for testicular germ cell tumors subtyping. Epigenomics.

[113] Ruggiero D., Nappo S., Nutile T., Sorice R., Talotta F., Giorgio E., Bellenguez C., Leutenegger A.-L., Liguori G.L., Ciullo M. (2015). Genetic variants modulating CRIPTO serum levels identified by genome-wide association study in Cilento isolates. PLoS Genet..

[114] Hass H.G., Vogel U., Scheurlen M., Jobst J. (2018). Use of Gene Expression Analysis for Discrimination of Primary and Secondary Adenocarcinoma of the Liver. Oncology.

[115] Ledda M., Megiorni F., Pozzi D., Giuliani L., D’Emilia E., Piccirillo S., Mattei C., Grimaldi S., Lisi A. (2013). Non ionising radiation as a non chemical strategy in regenerative medicine: Ca(2+)-ICR “In Vitro” effect on neuronal differentiation and tumorigenicity modulation in NT2 cells. PLoS ONE.

[116] Spike B.T., Kelber J.A., Booker E., Kalathur M., Rodewald R., Lipianskaya J., La J., He M., Wright T., Klemke R. (2014). CRIPTO/GRP78 signaling maintains fetal and adult mammary stem cells ex vivo. Stem Cell Rep..

[117] Miharada K., Karlsson G., Rehn M., Rörby E., Siva K., Cammenga J., Karlsson S. (2011). Cripto regulates hematopoietic stem cells as a hypoxic-niche-related factor through cell surface receptor GRP78. Cell Stem Cell.

[118] Gao L.R., Zhang N.K., Ding Q.A., Chen H.Y., Hu X., Jiang S., Li T.C., Chen Y., Wang Z.G., Ye Y. (2013). Common expression of stemness molecular markers and early cardiac transcription factors in human Wharton’s jelly-derived mesenchymal stem cells and embryonic stem cells. Cell Transplant..

[119] Hosseinpour B., Bakhtiarizadeh M.R., Khosravi P., Ebrahimie E. (2013). Predicting distinct organization of transcription factor binding sites on the promoter regions: a new genome-based approach to expand human embryonic stem cell regulatory network. Gene.

[120] Teshigawara R., Hirano K., Nagata S., Huang D., Tada T. (2019). Visualization of sequential conversion of human intermediately reprogrammed stem cells into iPS cells. Genes Cells Devoted Mol. Cell. Mech..

[121] Miharada K., Karlsson G., Rehn M., Rörby E., Siva K., Cammenga J., Karlsson S. (2012). Hematopoietic stem cells are regulated by Cripto, as an intermediary of HIF-1α in the hypoxic bone marrow niche. Ann. N. Y. Acad. Sci..

[122] Lonardo E., Parish C.L., Ponticelli S., Marasco D., Ribeiro D., Ruvo M., De Falco S., Arenas E., Minchiotti G. (2010). A small synthetic cripto blocking Peptide improves neural induction, dopaminergic differentiation, and functional integration of mouse embryonic stem cells in a rat model of Parkinson’s disease. Stem Cells Dayt. Ohio.

[123] Kang R., Zhou Y., Tan S., Zhou G., Aagaard L., Xie L., Bünger C., Bolund L., Luo Y. (2015). Mesenchymal stem cells derived from human induced pluripotent stem cells retain adequate osteogenicity and chondrogenicity but less adipogenicity. Stem Cell Res. Ther..

[124] Focà G., Iaccarino E., Focà A., Sanguigno L., Untiveros G., Cuevas-Nunez M., Strizzi L., Leonardi A., Ruvo M., Sandomenico A. (2019). Development of conformational antibodies targeting Cripto-1 with neutralizing effects in vitro. Biochimie.

[125] Gudbergsson J.M., Duroux M. (2020). An evaluation of different Cripto-1 antibodies and their variable results. J. Cell. Biochem..

[126] Adam A., Ras R., Bhattu A.S., Raman A., Perera M. (2017). “Researching the Research” in Prostate Cancer: A Comparative Bibliometric Analysis of the Top 100 Cited Articles in the Field of Prostate Cancer. Curr. Urol..

[127] (2019). R Core Team: R: A Language and Environment for Statistical Computing.

[128] R Studio (2016). R Studio: Integrated Development for R.

[129] Koseoglu M.A. (2016). Mapping the institutional collaboration network of strategic management research: 1980–2014. Scientometrics.

[130] Elango B., Rajendran P. (2012). Authorship trends and collaboration pattern in the marine sciences literature: a scientometric study. Int. J. Inf. Dissemin. Technol..

[131] Kumar S., Kumar S. Collaboration in research productivity in oil seed research institutes of India. Proceedings of the Fourth International Conference on Webometrics, Informetrics and Scientometrics & Ninth COLLNET Meeting, Proceedings of WIS 2008.

[132] Radhakrishnan S., Erbis S., Isaacs J.A., Kamarthi S. (2017). Novel keyword co-occurrence network-based methods to foster systematic reviews of scientific literature. PLoS ONE.

[133] Fellows I. (2018). Word Clouds. https://cran.r-project.org/web/packages/wordcloud/index.html.

[134] Falagas M.E., Pitsouni E.I., Malietzis G.A., Pappas G. (2008). Comparison of PubMed, Scopus, Web of Science, and Google Scholar: strengths and weaknesses. FASEB J..

