# Supplementary material for: A Multidisciplinary Review of the Roles of Cripto in the Scientific Literature Through a Bibliometric Analysis of its Biological Roles
Source: Cancers (Basel). 2020 Jun 5;12(6):1480. doi: 10.3390/cancers12061480 (PMC7352664; doi:10.3390/cancers12061480)
Supplement: Supplementary file 1 [file cancers-12-01480-s001.pdf]

Supplementary Materials: A Multidisciplinary Review of the Roles of Cripto in the Scientific Literature through a Bibliometric Analysis of its Biological Roles

Elisa Rodrigues Sousa, Eugenio Zoni, Sofia Karkampouna, Federico La Manna, Peter C. Gray, Marta De Menna and Marianna Kruithof-de Julio

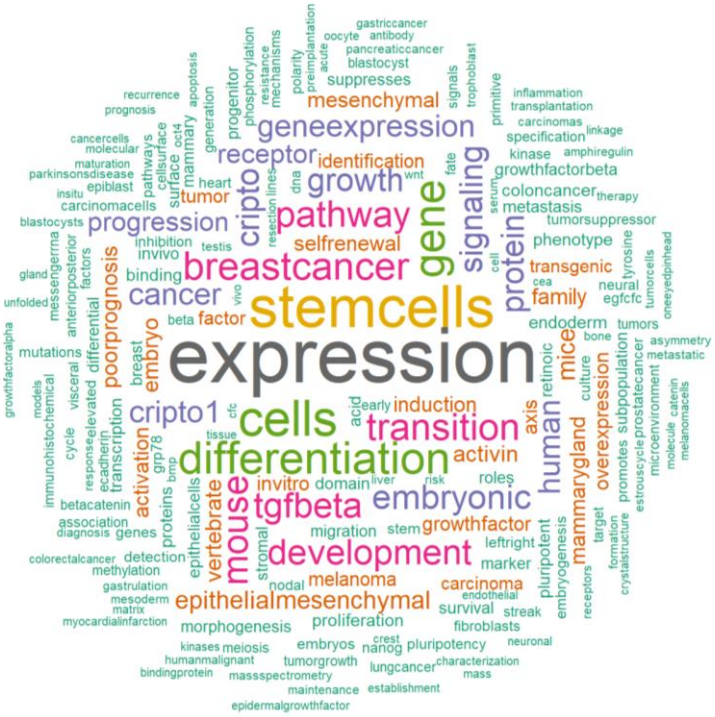

Figure S1. Wordcloud of keywords. Visual representation of most frequent keywords used on Cripto research field from 2010 and 2020.

© 2020 by the authors. Licensee MDPI, Basel, Switzerland. This article is an open access article distributed under the terms and conditions of the Creative Commons Attribution (CC BY) license (http://creativecommons.org/licenses/by/4.0/).
